# FoxO3a‐Mediated Modulation of PD‐L1 Expression and Inhibition by Dihydroartemisinin in Triple‐Negative Breast Cancer

**DOI:** 10.1111/jcmm.70947

**Published:** 2026-01-11

**Authors:** Xingan Xing, Zhiwei Zhou, Mohd Farhan, Xia Zhao, Shuai Li, Bingxi Lei, Jiankang Fang, Wenshu Zhou, Wenhua Zheng

**Affiliations:** ^1^ Pharmaceutical Science, Faculty of Health Sciences University of Macau Macau SAR China; ^2^ Guangdong‐Hong Kong‐Macao Joint Laboratory for New Drug Screening University of Macau Macau SAR China

**Keywords:** dihydroartemisinin, FoxO3a, immunotherapy, PD‐L1, TNBC, ubiquitination

## Abstract

Tumour immunotherapy targeting PD‐1/PD‐L1 shows promise, but the regulatory mechanisms of PD‐L1 and its small‐molecule modulators remain unclear. This study investigated FoxO3a‐mediated PD‐L1 regulation and the PD‐L1‐inhibitory role of dihydroartemisinin (DA) in triple‐negative breast cancer (TNBC). FoxO3a overexpression significantly increased PD‐L1 expression and impaired T cell‐mediated cytotoxicity, while knockdown exerted opposite effects in TNBC cells. Promoter motif analysis and dual‐luciferase assays revealed FoxO3a binding to the s155 site on the PD‐L1 promoter in MDA‐MB‐231 cells; mutation of s155 abolished this interaction. ChIP‐PCR confirmed FoxO3a binding to the PD‐L1 promoter at s155. Furthermore, DA, a clinical antimalarial, reduced PD‐L1 and FoxO3a levels, sensitising TNBC cells to T cell killing in TNBC cells. Mechanistically, DA enhanced IRE1/IKK phosphorylation, promoting FoxO3a Ser644 phosphorylation and ubiquitination. Crucially, s155 was required for DA‐induced PD‐L1 suppression in MDA‐MB‐231 cells. These findings demonstrate PD‐L1 as a direct transcriptional target of FoxO3a and identify DA as a potential TNBC therapeutic targeting the IRE1/IKK/FoxO3a/PD‐L1 axis.

## 1 Introduction

Triple‐negative breast cancer (TNBC), constituting approximately 10%–20% of breast cancers, is characterised by the absence of oestrogen receptors, progesterone receptors and human epidermal receptor 2 [1]. Clinically, TNBC patients tend to have poorer treatment outcomes compared to other breast cancer subtypes, due to its inherently aggressiveness and lack of well defined therapeutic targets [2]. Therefore, nonspecific chemotherapy remains the standard therapeutic approach for TNBC [3]. With the advancement of immunotherapy, it has been demonstrated that TNBC is also suitable for immunotherapeutic treatments. This is attributed to the presence of tumour immune infiltration, an increase in immunogenicity caused by mutational burden and higher genomic instability, as well as elevated levels of immune markers [4].

Programmed cell death receptor 1 (PD1) and its ligand PD‐L1 are a pair of well known immune checkpoints that are critical to maintaining balanced immune activity [5]. The interaction between PD‐L1 on cancer cells and PD1 on activated cytotoxic T lymphocytes infiltrating into tumours, triggers inhibitory signals in T lymphocyte activation, proliferation, cytokine production, and cytolytic activity, facilitating immune escape in cancer cells [6]. Thus, PD‐L1 or PD1 blocking agents, predominantly neutralising antibodies, have shown tremendous clinical benefits in various cancers [7]. TNBC, distinguished by higher PD‐L1 expression and increased CD8+ T‐cell infiltration, is considered suitable for PD‐L1 blocking therapy [8]. In 2019, atezolizumab (anti‐PD‐L1 antibody) plus nab‐paclitaxel became the first FDA‐approved immune checkpoint inhibitor for PD‐L1 positive TNBC treatment based on the results from the IMpassion130 trial [9]. However, the benefits of immunotherapeutic treatments using monoclonal antibodies are limited to a small part of patients. The effectiveness of a single anti‐PD‐L1 antibody is limited, achieving a response rate of only about 15%–30%, and safety concerns with this therapy have been less than satisfactory [10]. Therefore, understanding the molecular mechanism of PD‐L1 regulation in tumours is necessary for optimising the outcomes of PD‐L1 blocking therapy in TNBC [11].

FoxO3a, a member of the Forkhead Box O (FoxO) family of transcription factors, is regulated by post‐translational modifications, influencing its cytoplasmic‐nuclear localization, transcriptional activity and protein stability [12]. Typically, activation of PI3K/AKT by insulin signalling phosphorylates FoxO3a, resulting in an increase in the binding of FoxO3a and 14–3‐3 protein and its cytoplasmic localization [13]. Alternatively, IKK‐mediated phosphorylation of FoxO3a at Ser644 results in nuclear export and ubiquitination‐mediated degradation [14]. FoxO3a plays a vital role in regulating the transcription of genes associated with cell cycle progression, apoptosis, metastasis, angiogenesis and metabolism in cancers [15]. However, there is presently no evidence supporting PD‐L1 as a direct target of the transcription factor FoxO3a in TNBC.

Dihydroartemisinin (DA), a semi‐synthetic derivative of artemisinin, has been utilised as a clinical antimalarial drug for many years and boasts a well established safety profile. Beyond its primary antimalarial function, extensive research has highlighted additional therapeutic properties of artemisinin and its derivatives, including anti‐inflammatory, antiviral and neuroprotective effects [16, 17]. DA has also demonstrated anticancer effects in various tumours, including liver, ovarian, lung and breast cancer, by inhibiting cell proliferation, migration, and invasion, inducing apoptosis and inhibiting angiogenesis [18, 19]. Nevertheless, the potential role of DA as a regulator of PD‐L1 expression in TNBC and its consequent antitumor effects has yet to be explored.

In this study, our aim was to investigate the regulatory role of the transcription factor FoxO3a on PD‐L1 expression in TNBC, and to explore the potential of DA in modulating PD‐L1 levels and enhancing T‐cell‐mediated antitumor immunity against TNBC cells.

## 2 Materials and Methods

### 2.1 Cell Culture and Materials

Human cancer cells MDA‐MB‐231 and MDA‐MB‐436 were generously provided by the laboratories of Professor Hang Fai KWOK and Professor Chuxia DENG, respectively. These cells were cultured in DMEM supplemented with 10% foetal bovine serum (FBS), 100 μg/mL streptomycin, and 100 units/mL penicillin, and maintained at 37°C with 5% CO_2_. DA was obtained from Meilunbio Company (Dalian, China).

### 2.2 Transient Gene Overexpression and Knockdown With Plasmid and *siRNA*

To achieve transient overexpression of FoxO3a, a human *FoxO3a* (NM_001106395) cDNA was synthesised and cloned into a pcDNA3.1 vector (Genechem Biotechnology, Shanghai, China). For transient knockdown experiments, small interfering RNAs (siRNAs) targeting *FoxO3a* or *PD‐L1* were used. The siRNA sequences targeting *FoxO3a* were 5′‐UGACAGAAUUCGACAAGGCAC‐3′ and 5′‐GUGCCUUGUCGAAUUCUGUCA‐3′, while the siRNAs targeting *PD‐L1* were 5′‐GCAGUGACCAUCAAGUCCUTT‐3′, 5′‐AGGACUUGAUGGUCACUGCTT‐3′. Plasmid or iRNA transfections were performed using Lipofectamine 3000 (Invitrogen, Carlsbad, CA, USA) according to the manufacturer's protocols. Approximately 48 h post‐transfection, cells were harvested for subsequent analyses or experiments.

### 2.3 Western Blotting

Cell lysis was performed using RIPA buffer with PMSF and a protease inhibitor cocktail. Protein levels were detected by western blotting as previously described [20]. Antibodies used included T‐FoxO3a (Cell Signalling Technology, Danvers, MA, USA, 2497), p644‐FoxO3a (Signalway antibody, College Park, MD, USA, 12978), PD‐L1 (Abcam, Cambridge, UK, 238697), GAPDH (Cell Signalling Technology, 3683), β‐Tubulin (Cell Signalling Technology, 2128), T‐IKKβ (Cell Signalling Technology, 8943) and p‐IKKα/β (Ser176/180) (Cell Signalling Technology, 2694).

### 2.4 Immunofluorescence and Immunohistochemical Assay

Immunofluorescence and immunohistochemistry analyses were performed following the protocol previously described for our laboratory [21]. For immunofluorescence, cells were fixed with 4% Paraformaldehyde (PFA), blocked with 10% BSA for 1 h at room temperature, and incubated overnight with anti‐FoxO3a (Cell Signalling Technology, 2497) and anti‐PD‐L1 (Abcam, 238697) primary antibodies. After washing, cells were incubated with corresponding secondary antibodies, and images were captured using a Nikon A1R confocal microscope. For immunohistochemistry, paraffin‐embedded tissues were sectioned (4 μm). After rehydration and antigen retrieval, endogenous peroxidase activity was blocked with 10% BSA. Primary antibodies against FoxO3a (Signalway antibody, 29036) or PD‐L1 (Abcam, 238697) were incubated overnight at 4°C. Sections were then washed, incubated with a secondary antibody, and subjected to DAB colour development. Slides were examined and photographed using an EVOS M7000 imaging system.

### 2.5 Total RNA Purification and Real‐Time Quantitative PCR (qPCR) Analysis

Total RNA extraction and cDNA synthesis were performed as described previously [20]. qPCR was performed on an Applied Biosystems 7500 Fast system using SYBR Green Real‐Time PCR Master Mixes (Thermal Fisher Scientific, Wilmington, Massachusetts, USA). Data were analysed using the comparative Ct method and normalised to the internal control GAPDH. Primer sequences were as follows: human GAPDH: forward, 5′‐AATCCCATCACCATCTTCC‐3′, reverse, 5′‐GAGTCCTTCCACGATACCAA‐3′; human PD‐L1: forward, 5′‐GGACAAGCAGTGACCATCAAG‐3′, reverse, 5′‐CCCAGAATTACCAAGTGAGTCCT‐3′.

### 2.6 PD1 and PD‐L1 Binding Assay

Cells in each group were fixed in 4% PFA for 15 min, incubated with recombinant human PD1 Fc protein (R&D Systems, Minneapolis, MN, USA) for 1 h, and then incubated with anti‐human Alexa Fluor 488 secondary antibodies (Thermal Fisher Scientific) for 1 h at room temperature. Nuclei were stained with DAPI, and cells were imaged using a Nikon A1R confocal microscope.

### 2.7 T Cell‐Mediated Tumour Cell Killing Assay

Human peripheral blood mononuclear cells (STEMCELL Technologies, Vancouver, BC, Canada) were cultured in ImmunoCult‐XF T‐cell expansion medium with ImmunoCult Human CD3/CD28/CD2 T‐cell activator (STEMCELL Technologies) and 10 ng/mL human IL‐2 (STEMCELL Technologies) for 7 days. Cancer cells were seeded in 6‐well plates overnight, and activated T cells were added, incubating for another 48 h in the presence or absence of DA in DMEM/F12 medium supplemented with anti‐CD3 antibody (100 ng/mL, Thermo Fisher Scientific) and IL‐2 (10 ng/mL). The ratio of cancer cells to T cells was 1:3. Finally, suspension cells including T cells and cell debris were gently washed twice with PBS. Living cells that remained viable and adherent were fixed in absolute methanol for 15 min at room temperature, following staining with 0.5% crystal violet. The stained cells were imaged using a Celigo Image Cytometer (Nexcelom, Lawrence, Massachusetts, USA) and then quantified by a spectrometer at OD (570 nm).

### 2.8 Transient Luciferase Reporter Assays

A 1.5‐kb fragment (−1500 to +1 bp) from the human PD‐L1 gene promoter region was identified using USUC bioinformatics tools and cloned into the pGL3‐basic plasmid (Promega, Madison, WI, USA), which contains the firefly luciferase reporter gene. The resulting plasmid was designated pGL3‐WT. Within this region, several potential FoxO3a binding motifs (AAACA) were identified. To assess the functional importance of each site, site‐directed mutagenesis was performed using the Phusion Site‐Directed Mutagenesis Kit (Thermo Fisher Scientific), converting AAACA to TGCAT at each predicted site. This generated four mutant reporter plasmids: pGL3‐s1456Mu, pGL3‐s936Mu, pGL3‐s167Mu and pGL3‐s155Mu. For luciferase assays, cells were plated in 48‐well plates and allowed to adhere overnight. On the following day, cells were transfected with the indicated reporter plasmids along with a Renilla luciferase control plasmid, using Lipofectamine 2000 (Invitrogen) according to the manufacturer's instructions. After 36 h, cells were washed with PBS and lysed for luciferase measurement. Firefly and Renilla luciferase activities were quantified using the Dual‐Luciferase Reporter Assay System (Promega). Normalised luciferase activity was calculated by dividing the firefly luciferase activity by the Renilla luciferase activity and expressing this ratio relative to the activity of the pGL3‐basic control plasmid.

### 2.9 ChIP‐PCR Assay

Cells for each chromatin immunoprecipitation (ChIP) were performed by the SimpleChIP Plus Enzymatic Chromatin IP Kit (Magnetic Beads) following the instructions from the Cell Signalling Technology Company. Cells were fixed with 1% formaldehyde and quenched with glycine. After centrifugation, cell pellets suspended in lysis buffer were digested by micrococcal nuclease and sonicated in 3 sets of 30‐s pulses using an ultrasonic homogeniser. The soluble chromatin (15 μg) was immunoprecipitated by incubating cell lysates with 3 μg anti‐FoxO3a antibody (Abcam, 12126) or rabbit IgG overnight at 4°C with rotation. Protein G magnetic beads (30 μL) were used to pellet immune complexes. Chromatin was eluted with elution buffer for 30 min at 65°C with gentle vortexing. The samples were reversed cross‐linked by adding 6 μL of 5 M NaCl and 2 μL of proteinase K, followed by incubation for 2 h at 65°C. Purified DNA was used for PCR amplification using primers targeting the human PD‐L1 gene promoter region: forward: 5′‐CAGATGTTGGCTTGTTGTAA‐3′; reverse: 5′‐GTATCTAGTGTTGGTGTCCTA‐3′.

### 2.10 MTT Assay

Cell viability was measured by MTT assay. Briefly, cells (5 × 10^3^ cells/well) were seeded in 96‐well plates overnight and treated with DA for another 24 h. The MTT assay was performed following a previously described protocol [20]. Cell viability is shown as a percentage of the control group.

### 2.11 Animal Studies

Six‐week‐old female Balb/c nude mice were obtained from the animal research core, University of Macau. The animal protocol was approved by the Ethics Committee of the University of Macau (protocol No.: UMARE‐015‐2017). MDA‐MB‐231 cells (1 × 10^6^) were subcutaneously injected into the mice. Six days after cell inoculation, the mice received daily injections of either vehicle DMSO (CTRL group, *n* = 5) or 40 mg/kg DA (DA group, *n* = 5). Tumour size was measured every 3 days, and tumour volume was calculated using the equation (length × width × width)/2. After the experiment, the mice were anaesthetised, sacrificed, and tumours were removed and weighed. Then, samples were frozen in liquid nitrogen and paraffin‐embedded for further analysis.

### 2.12 Immunoprecipitation

Cells cultured in 10‐cm dishes were washed with precooled PBS and collected in 500 μL of lysis buffer with DTT and Halt Protease Inhibitor Cocktail (Invitrogen). The cell lysate was precleared with 50 μL of protein G/A agarose slurry (Thermo Fisher Scientific). Proteins were immunoprecipitated overnight with 3 μg of specific antibodies against FoxO3a (Abcam, 12126), TRAF2 (Signalway antibody, 49196), or rabbit IgG. The immune complexes were captured by 30 μL of protein G/A agarose slurry and then washed three times with cell lysis buffer. The proteins were eluted in 4× SDS loading buffer and boiled at 95°C for 15 min. Target protein levels were analysed by western blotting.

### 2.13 Statistical Analysis

All data are presented as the mean ± SD from three independent experiments. Statistical analyses were analysed using SPSS (Ver. 19). One‐way ANOVA, combined with a post hoc test, was used to compare multiple groups of independent samples. The independent sample *t* test was performed to compare mean values. A *p* value < 0.05 was regarded as statistically significant.

## 3 Results

### 3.1 FoxO3a Regulated PD‐L1 Expression and Cell Sensitivity to Activated T Cells in TNBC Cells

**FIGURE 1:**
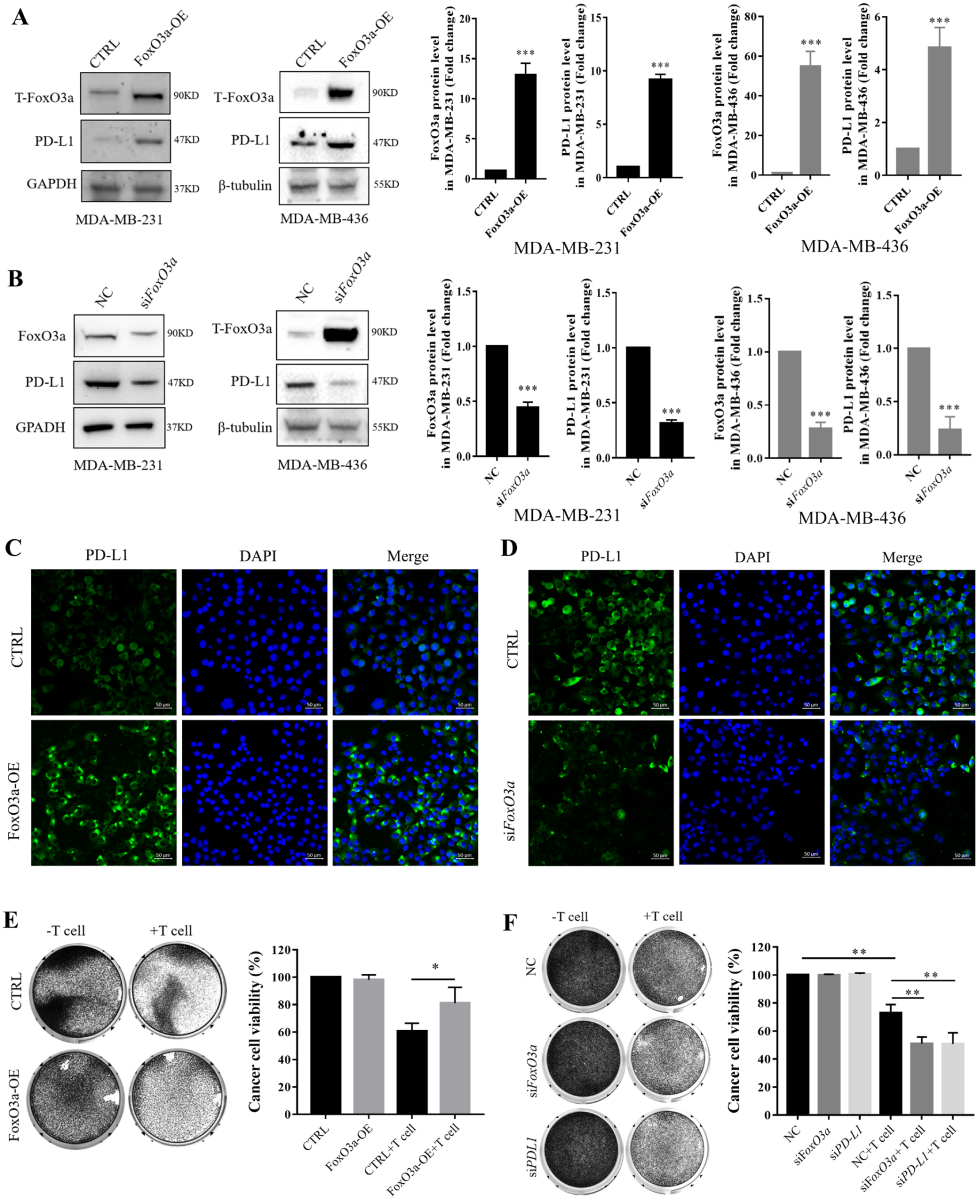
FoxO3a regulates PD‐L1 expression in TNBC. (A) Western blotting (Left) and quantification (right) of FoxO3a and PD‐L1 protein levels following FoxO3a overexpression in MDA‐MB‐231 and MDA‐MB‐436 cells. (B) Western blotting (Left) and quantification (right) of FoxO3a and PD‐L1 protein levels following FoxO3a knockdown in MDA‐MB‐231 and MDA‐MB‐436 cells. Representative immunofluorescence images of PD‐L1 levels in FoxO3a overexpressed (C) and knockdown (D) MDA‐MB‐231 cells. (E) Crystal violet staining images (left) and normalised results (right) of T cell‐mediated cancer cell killing assay results in FoxO3a overexpressed MDA‐MB‐231 cells. (F) Crystal violet staining images (left) and normalised results (right) T cell‐mediated cancer cell killing assay results in FoxO3a or PD‐L1 knockdown MDA‐MB‐231 cells. Statistical significance was determined using *p < 0.05, **p < 0.01 and ***p < 0.001. All error bars are expressed as mean ± SD of three independent experiments. CTRL, negative control vector plasmid; NC, negative control siRNA; OE, overexpression plasmid; si*FoxO3a*, siRNAs targeting *FoxO3a*; *s*i*PD‐L1*, siRNAs targeting *PDl‐L1*; TNBC, Triple‐negative breast cancer.

To test whether FoxO3a affects PD‐L1 levels, we generated FoxO3a‐overexpressing human TNBC cell lines, MDA‐MB‐231 and MDA‐MB‐436. Western blotting revealed a pronounced increase in PD‐L1 protein levels in the FoxO3a overexpression (FoxO3a‐OE) group (Figure 1A). Conversely, FoxO3a knockdown via FoxO3a‐specific siRNA significantly attenuated the PD‐L1 protein level in MDA‐MB‐231 and MDA‐MB‐436 cells (Figure 1B). Immunofluorescence further confirmed FoxO3a‐induced PD‐L1 expression in MDA‐MB‐231 cells, with the fluorescence signal representing PD‐L1 levels being upregulated in the FoxO3a‐OE group and significantly decreased in FoxO3a‐knockdown MDA‐MB‐231 cells (Figure 1C,D). Next, we explored the functional significance of FoxO3a overexpression in the anticancer immune response. MDA‐MB‐231 cells were co‐cultured with activated T cells in vitro, as described in the manuscript methods. FoxO3a overexpression rendered MDA‐MB‐231 cells more resistant to activated T cells in T‐cell‐mediated tumour cell killing (Figure 1E). Conversely, knockdown of FoxO3a sensitised MDA‐MB‐231 cells to activated T cells (Figure 1F). Together, these findings show that FoxO3a positively regulated PD‐L1 expression in TNBC cells, contributing to cancer cell immune escape in vitro.

### 3.2 FoxO3a Transcriptionally Activated PD‐L1 Through Direct Binding to the PD‐L1 Gene Promoter

**FIGURE 2:**
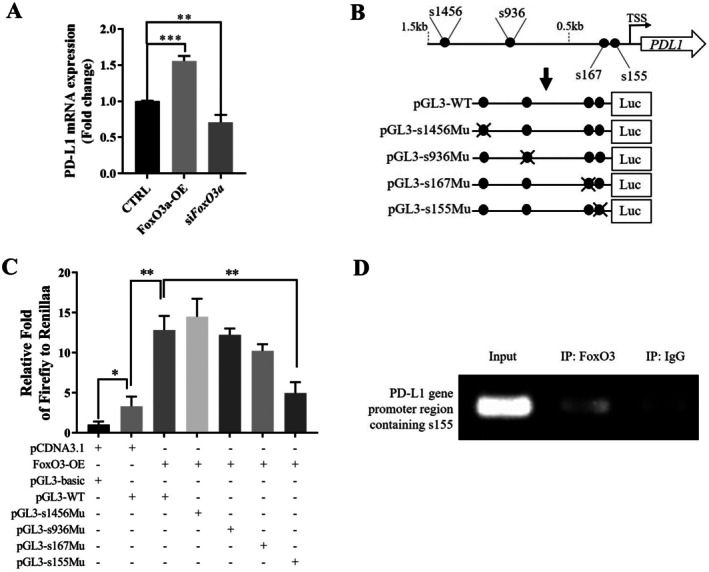
FoxO3a directly binds to the PD‐L1 gene promoter in MDA‐MB‐231 cells. (A) qPCR analysis of PD‐L1 mRNA expression in FoxO3a overexpressed and knockdown MDA‐MB‐231 cells. (B) Schematic representation of the human PD‐L1 gene promoter region and the four identified FoxO3a binding sites (Top). The wild‐type and site‐specific mutant promoter sequences were cloned into pGL3 reporter plasmids (down). (C) Dual‐luciferase reporter assays comparing the activities of WT and mutant PD‐L1 promoter in MDA‐MB‐231 cells co‐transfected with the indicated plasmids. (D) ChIP‐PCR analysis using a FoxO3a‐specific antibody to amplify the PD‐L1 gene promoter region (−289 to −110) in MDA‐MB‐231 cells. Statistical significance was determined using *p < 0.05, **p < 0.01 and ***p < 0.001. All error bars are expressed as mean ± SD of three independent experiments. ChIP, chromatin immunoprecipitation; CTRL, negative control vector plasmid or negative control siRNA; IP, immunoprecipitation; OE, overexpression plasmid; s1456Mu, Mutation at position 1456 in the PD‐L1 promoter element; s155Mu, Mutation at position 155 in the PD‐L1 promoter element; s167Mu, Mutation at position 167 in the PD‐L1 promoter element; s936Mu, Mutation at position 936 in the PD‐L1 promoter element; TSS: Transcription start site; WT, wild‐type PD‐L1 promoter element.

Considering the established role of FoxO3a as a transcription factor, we first examined whether FoxO3a regulates PD‐L1 at the transcriptional level in MDA‐MB‐231 cells. Overexpression of FoxO3a significantly increased PD‐L1 mRNA levels, while FoxO3a knockdown reduced PD‐L1 mRNA expression (Figure 2A). To identify the specific FoxO3a‐regulating region within the PD‐L1 promoter, we conducted a bioinformatic analysis and identified four potential FoxO3a binding sites (s1456, s936, s167 and s155) within the −1500 bp to +1 bp region of the PD‐L1 promoter (Figure 2B). A luciferase reporter construct containing the wild‐type PD‐L1 promoter (pGL3‐WT) demonstrated basal promoter activity, which was enhanced under endogenous FoxO3a conditions (Figure 2C, panels 1–2). Further overexpression of FoxO3a led to a marked increase in PD‐L1 promoter‐driven luciferase activity, indicating that FoxO3a positively regulates PD‐L1 transcription (Figure 2C, panels 2–3).

To determine which of the identified binding sites mediates FoxO3a's regulatory effect, we generated four reporter plasmids, each carrying a mutation in one of the potential FoxO3a binding sites (Figure 2B). Among these variants, only mutation of the s155 site abolished the FoxO3a‐induced increase in PD‐L1 promoter activity (Figure 2C, panels 3–7). ChIP assays using a FoxO3a‐specific antibody confirmed that FoxO3a directly binds to the PD‐L1 promoter at the s155 site (Figure 2D). Collectively, these results demonstrate that FoxO3a directly stimulates PD‐L1 transcription via the s155 site in the PD‐L1 promoter in TNBC cells.

### 3.3 DA Reduced PD‐L1 Expression in TNBC Cells

**FIGURE 3:**
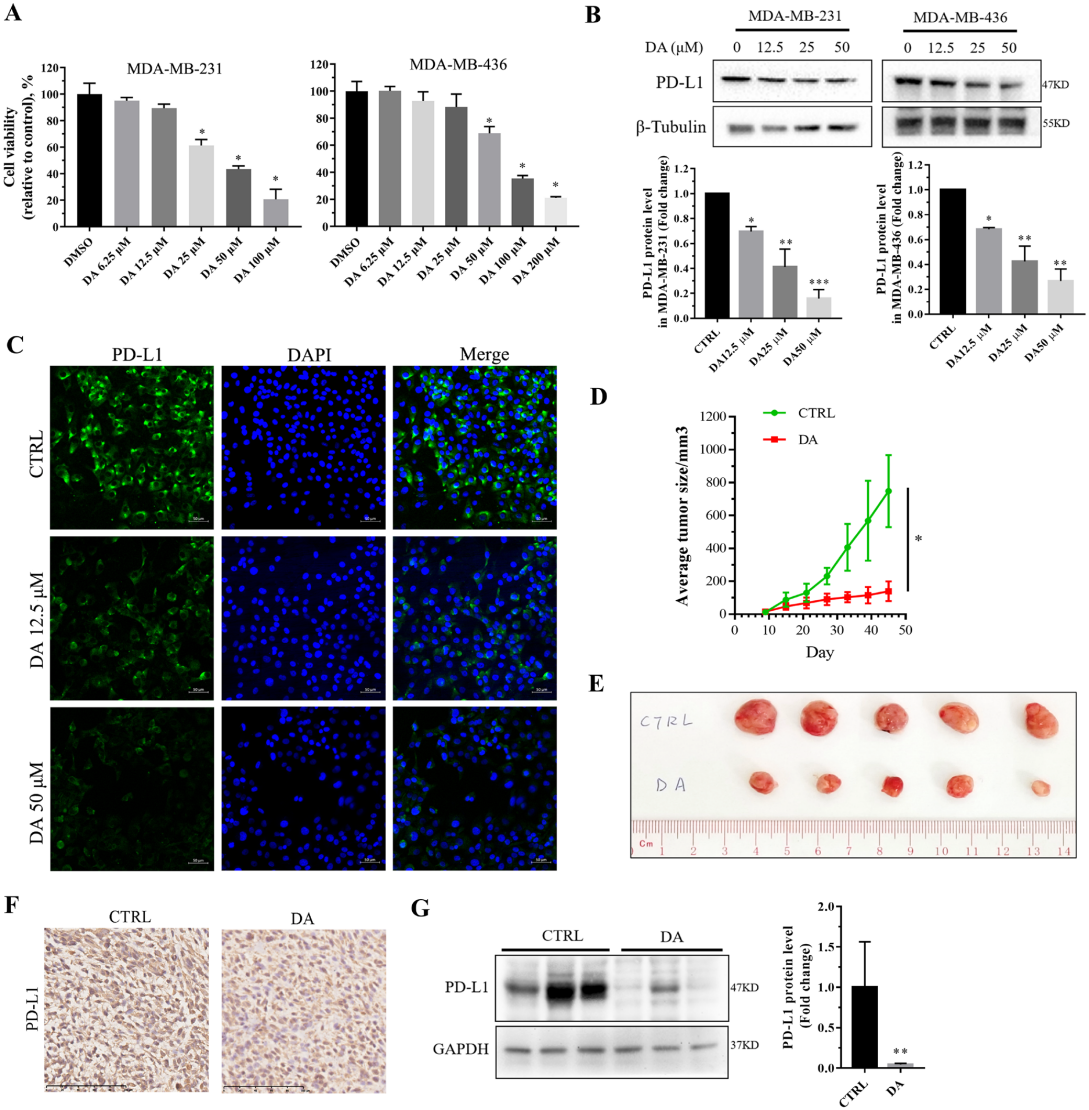
DA treatment decreases PD‐L1 expression in TNBC cells in vitro and in vivo. MDA‐MB‐231 or MDA‐MB‐436 cells were treated with or without DA for 24 h. (A) Cell viability results in MDA‐MB‐231 (left) and MDA‐MB‐436 (right) cells treated with different concentrations of DA. (B) Western blotting analysis and quantification of PD‐L1 protein levels in MDA‐MB‐231 and MDA‐MB‐436 cells treated with different concentrations of DA. (C) Representative images of immunofluorescence staining of PD‐L1 levels in MDA‐MB‐231 cells treated with DA. (D) Quantification of tumour volume in MDA‐MB‐231 xenograft nude mice treated with 40 mg/kg DA. (E) Representative images of dissected tumours at the end point. (F) Representative images of PD‐L1 immunohistochemical staining in tumours. (G) Western blotting and quantification of PD‐L1 levels in tumours from CTRL and DA groups. Statistical significance was determined using *p < 0.05, **p < 0.01 and ***p < 0.001. All error bars are expressed as mean ± SD of three independent experiments. CTRL, negative control; DA, dihydroartemisinin.

The anticancer effects of DA have primarily focused on cancer cell proliferation, apoptosis and cytotoxicity, with limited studies exploring its impact on anticancer immunity and the mechanisms. Consistent with previous reports [22], DA treatment at up to 50 μM significantly induced cell death in MDA‐MB‐231 and MDA‐MB‐436 cells (Figure 3A). To study whether DA can regulate the expression of PD‐L1 in TNBC, MDA‐MB‐231 and MDA‐MB‐436 cells were treated with different concentrations of DA (12.5–50 μM). Western blotting showed that DA treatment significantly reduced PD‐L1 protein levels in MDA‐MB‐231 and MDA‐MB‐436 cells in a concentration‐dependent manner (Figure 3B). Immunofluorescence analysis of cellular PD‐L1 also showed a significant decrease in PD‐L1 in MDA‐MB‐231 cells under DA treatment (Figure 3C). We then studied the antitumor effect of DA in vivo. MDA‐MB‐231 xenograft nude mice were dosed daily with 40 mg/kg DA or vehicle. The dosage of DA was aligned with previous in vivo studies investigating its effects [23]. Compared with the control group, DA administration for 45 days significantly retarded tumour growth (Figure 3D,E). More notably, immunohistochemical and western blotting results showed that PD‐L1 protein levels in tumours were significantly decreased in the DA group (Figure 3F,G). These results indicate that DA can significantly inhibit PD‐L1 expression in vitro in TNBC cells and in vivo in animal models.

### 3.4 DA Enhanced MDA‐MB‐231 Cell Sensitivity to Activated T‐Cell‐Mediated Cell Killing In Vitro

**FIGURE 4:**
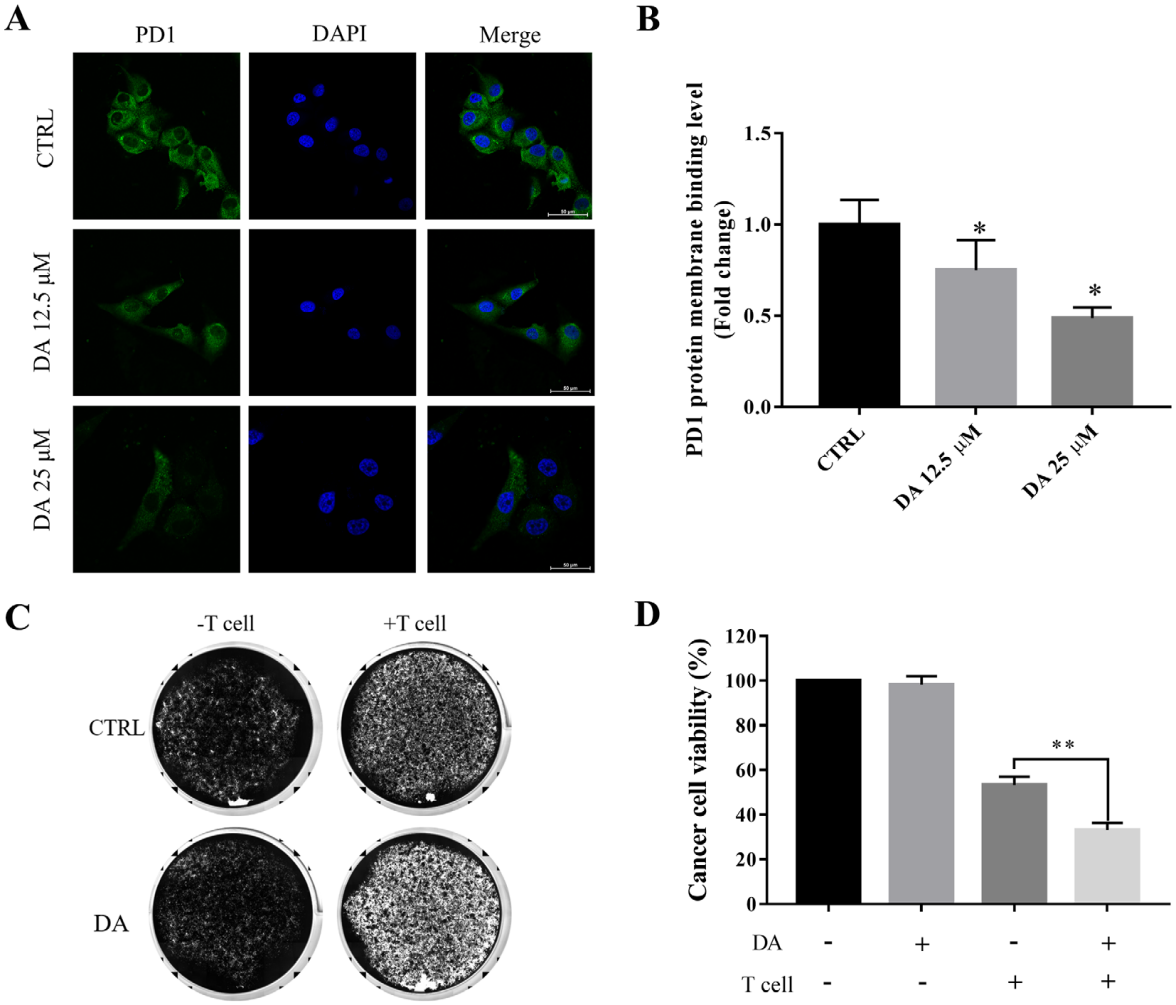
DA treatment sensitises MDA‐MB‐231 cells to activated T cells. Representative images (A) and quantification (B) of green fluorescence‐labelled PD1/Fc on DA‐treated MDA‐MB‐231 cells for 24 h. Representative images (C) and quantification (D) of crystal violet‐stained live cancer cells in T cell‐mediated cancer cell killing assay. MDA‐MB‐231 cells were then co‐cultured with activated T cells for 48 h with or without DA (12.5 μM) and subjected to crystal violet staining. Statistical significance was determined using *p < 0.05 and **p < 0.01. All error bars are expressed as mean ± SD of three independent experiments. Abbreviations: DA, dihydroartemisinin; CTRL, negative control.

Then, we measured the binding ability of PD‐L1 on the surface of tumour cells to PD1 following DA treatment. MDA‐MB‐231 cells were incubated with recombinant human PD1 Fc chimeric protein after treatment with or without DA. The results showed that DA treatment led to a decrease in the binding of PD1 to the tumour cells (Figure 4A,B), which was consistent with the reduction in PD‐L1 on the tumour surface by DA treatment. To test whether the reduction in PD‐L1 improves the antitumor effect, we next performed an in vitro T‐cell‐mediated tumour cell killing test. MDA‐MB‐231 cells were treated with DA and then incubated with activated T cells. Crystal violet staining (live cells, Figure 4C,D) showed that MDA‐MB‐231 cells co‐cultured with activated T cells significantly induced cell death. In other words, MDA‐MB‐231 cells treated with DA were more sensitive to activated T cells, and DA treatment significantly enhanced the killing effect of activated T cells compared with the control. Together, these findings suggest that DA enhances the sensitivity of TNBC cells to activated T cells in vitro, which is supported by the decrease in TNBC surface PD‐L1 levels and the binding of PD1/PD‐L1 by DA treatment.

### 3.5 DA Decreased FoxO Family Transcription Factor FoxO3a Protein Levels in TNBC Cells

**FIGURE 5:**
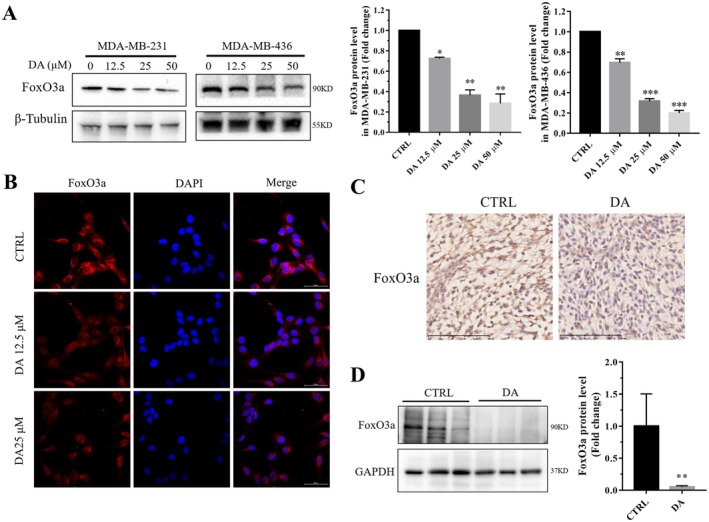
DA treatment decreases FoxO3a levels in TNBC. MDA‐MB‐231 and MDA‐MB‐436 cells were exposed to DA for 24 h. (A) Western blotting (left) and quantification (right) of FoxO3a protein levels. (B) Representative images of FoxO3a immunofluorescence staining in MDA‐MB‐231 cells treated with or without DA for 24 h. (C) Representative images of FoxO3a immunohistochemical staining in MDA‐MB‐231 xenograft nude mice tumours. (D) Western blotting analysis and quantification of FoxO3a levels in MDA‐MB‐231 xenograft nude mice tumours. Statistical significance was determined using *p < 0.05, **p < 0.01 and ***p < 0.001. All error bars are expressed as mean ± SD of three independent experiments. CTRL, negative control; DA, dihydroartemisinin.

FoxO3a is a FoxO subfamily member transcription factor involved in cell proliferation, apoptosis, cancer growth, migration and other physiological and pathological processes [24]. Interestingly, western blotting showed that FoxO3a protein levels in MDA‐MB‐231 and MDA‐MB‐436 cells were significantly reduced by DA treatment (Figure 5A). To support this finding, immunofluorescence staining also showed a decrease in FoxO3a in MDA‐MB‐231 cells (Figure 5B). Moreover, FoxO3a levels in tumours of MDA‐MB‐231 xenograft nude mice, analysed by both immunohistochemistry and western blotting, showed a decrease in the DA administration group (Figure 5C,D). These results demonstrate that DA could reduce FoxO3a protein levels in TNBC.

### 3.6 DA Inhibited PD‐L1 mRNA Expression by Decreasing FoxO3a Binding to the PD‐L1 Gene Promoter at s155 in MDA‐MB‐231 Cells

**FIGURE 6:**
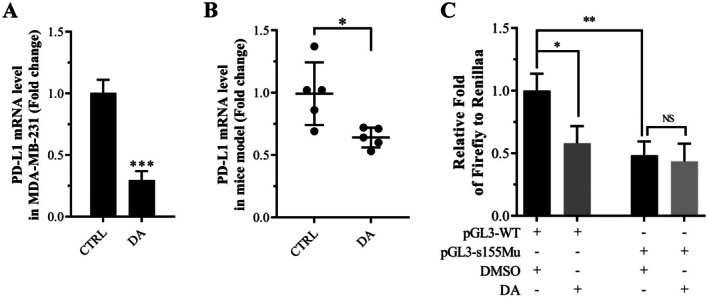
DA treatment inhibits PD‐L1 mRNA expression by reducing FoxO3a binding to the PD‐L1 gene promoter at s155 in MDA‐MB‐231 cells. PD‐L1 mRNA expression levels in DA‐treated MDA‐MB‐231 cells (A) and MDA‐MB‐231 xenograft nude mice tumours (B). (C) Dual luciferase assay analysis of pGL3‐WT and pGL3‐s155Mu‐expressing MDA‐MB‐231 cells under DA treatment. Statistical significance was determined using *p < 0.05, **p < 0.01 and ***p < 0.001. All error bars are expressed as mean ± SD of three independent experiments. CTRL, negative control; DA, dihydroartemisinin; s155Mu, mutation at position 155 in the PD‐L1 promoter element; WT, wild‐type PD‐L1 promoter element.

We further investigated whether FoxO3a‐mediated regulation of PD‐L1 is involved in the inhibitory effect of DA on PD‐L1. The mRNA levels of PD‐L1 in both MDA‐MB‐231 cells and a nude mouse model were significantly reduced following DA treatment (Figure 6A,B). Then, MDA‐MB‐231 cells were transfected with PD‐L1 promoter reporter plasmids pGL3‐WT or pGL3‐s155Mu, and treated with or without DA. The results from dual luciferase assays indicated a significant reduction in the activity of the wild‐type PD‐L1 gene promoter by DA (Figure 6C), suggesting that the modulation of PD‐L1 gene transcription by DA treatment could be responsible for the downregulation of PD‐L1 protein levels. Furthermore, the mutation of s155 abolished the inhibitory effect of DA on PD‐L1 gene promoter activity (Figure 6C). This implies that the binding of FoxO3a to the PD‐L1 gene promoter at s155 is necessary for the DA‐induced suppression of PD‐L1 expression in MDA‐MB‐231 cells.

### 3.7 DA Increased the Phosphorylation and Ubiquitination of FoxO3a Through Activation of IKK in MDA‐MB‐231 Cells

**FIGURE 7:**
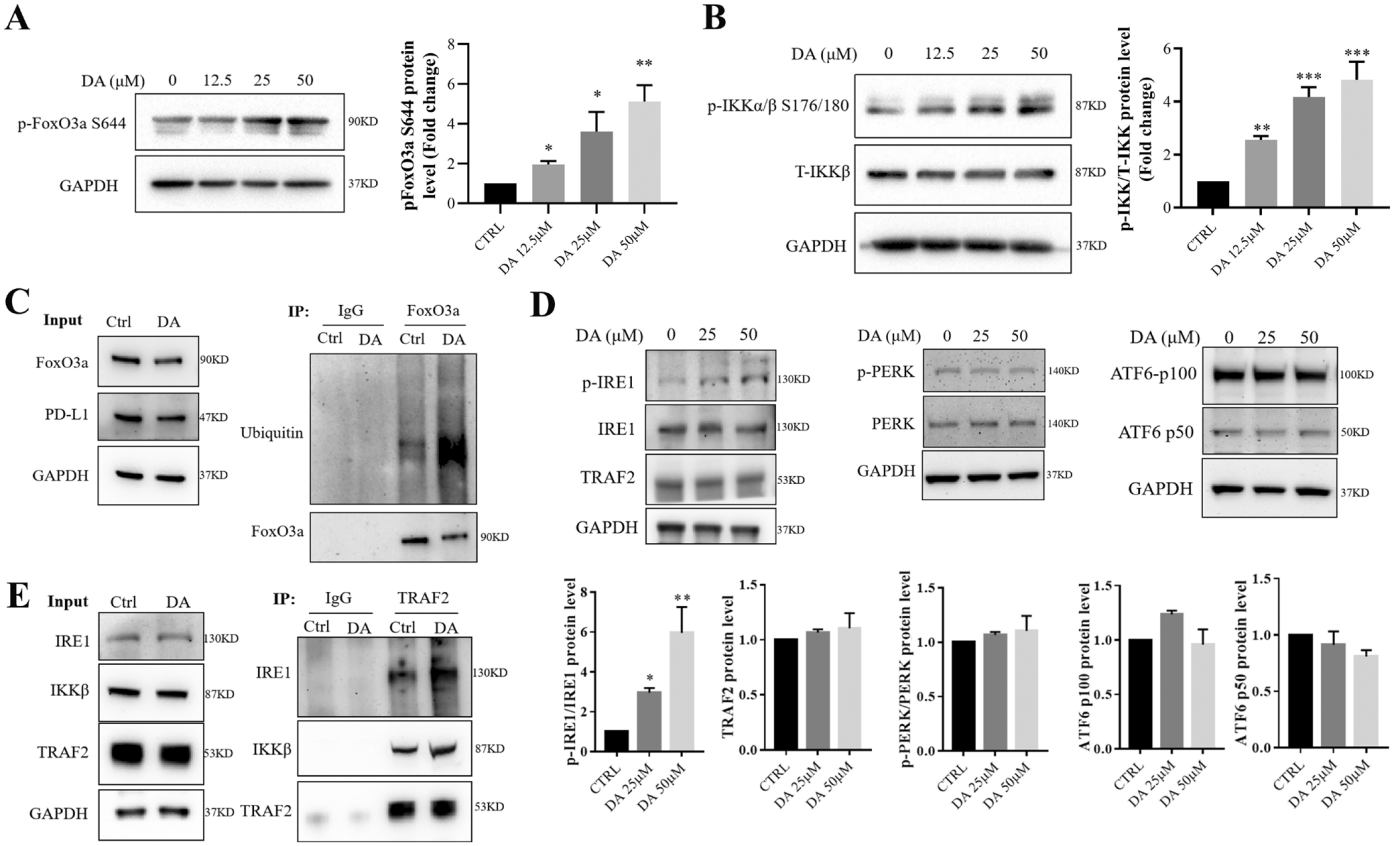
DA modulates the IRE1/TRAF2/IKK/FoxO3a signalling pathway in MDA‐MB‐231 cells. (A) Western blotting (left) and quantification (right) of FoxO3a Ser644 phosphorylation levels in MDA‐MB‐231 cells treated with or without DA for 24 h. (B) Western blotting (left) and quantification (right) of p‐IKK and T‐IKK protein levels in MDA‐MB‐231 cells treated with or without DA for 24 h. (C) Ubiquitination of FoxO3a in MDA‐MB‐231 cells treated with or without DA for 24 h. (D) Western blotting and quantification of p‐IRE1, T‐IRE1 PERK, T‐PERK, ATF6‐p100 and ATF6‐p50 protein levels in MDA‐MB‐231 cells treated with or without DA for 24 h. (E) Co‐immunoprecipitation assay measuring the interaction of IRE1, IKKβ and TRAF2 in MDA‐MB‐231 cells treated with or without DA for 24 h. Statistical significance was determined using *p < 0.05, **p < 0.01 and ***p < 0.001. All error bars are expressed as mean ± SD of three independent experiments. CTRL, negative control; DA, dihydroartemisinin; IP, immunoprecipitation.

The phosphorylation of FoxO3a plays an essential role in its subcellular localization, transcriptional activity and stability [24]. Specifically, phosphorylation of FoxO3a at Ser644 by IκB kinase (IKK) promotes FoxO3a cytoplasmic localization and, eventually, ubiquitination‐mediated degradation [14]. To see if these processes occur with DA treatment, we examined the phosphorylation of IKK and FoxO3a at Ser644, as well as the ubiquitination‐mediated degradation of FoxO3a in DA‐treated cells. Figure 7A,B showed a significant increase in the phosphorylation levels of FoxO3a at Ser644 and IKK in MDA‐MB‐231 cells treated with DA. Additionally, immunoprecipitation using a FoxO3a‐specific antibody revealed an increase in the ubiquitin level associated with FoxO3a, concomitant with a reduction in total FoxO3a in DA‐treated MDA‐MB‐231 cells (Figure 7C). These findings showed that DA treatment induced the phosphorylation of IKK, leading to ubiquitination‐mediated degradation of FoxO3a.

### 3.8 DA Stimulated the Phosphorylation of IRE1/IKK and Their Association With TRAF2

The mechanism through which DA stimulates the phosphorylation of IKK in MDA‐MB‐231 cells remains unclear. Previous studies have reported that IRE1α can form a complex with IKK via TRAF2, resulting in the phosphorylation and activation of IKK during endoplasmic reticulum (ER) stress [25]. In light of this, we investigated the effect of DA on the phosphorylation of three key proteins: IRE1, PERK and ATF6 in the ER stress pathway. Western blotting analysis showed a significant increase in IRE1 phosphorylation following DA treatment, while PERK and full‐length/cleaved ATF6 phosphorylation remained unaffected (Figure 7D). Furthermore, we conducted a co‐immunoprecipitation assay using a TRAF2‐specific antibody. Western blotting analysis with IRE1 or IKK antibodies demonstrated the presence of IRE1 and IKK proteins in the precipitate, with DA treatment enhancing the levels of IRE1 and IKK proteins in anti‐TRAF2 immunoprecipitates (Figure 7E). Together, these results indicated that upon DA treatment, IRE1 was activated by phosphorylation and formed an interaction with TRAF2 and IKK, leading to the activation/phosphorylation of IKK.

## 4 Discussion

Immune checkpoint inhibitors offer a promising avenue in TNBC therapy. PD‐L1, an important inhibitory molecule in tumour immunosurveillance, represents a target for immune checkpoint inhibitors [5]. Our study demonstrated PD‐L1 as a new direct downstream target of the transcription factor FoxO3a in TNBC. FoxO3a up‐regulated the expression of PD‐L1 via its binding to the PD‐L1 gene promoter. We also revealed that DA, a clinical antimalarial drug, effectively inhibited PD‐L1 expression through regulating IRE1/IKK/FoxO3a signalling, thereby enhancing antitumor immunity in TNBC.

Regulation of PD‐L1 expression at the cell surface is a complex process involving genomic aberrations, transcriptional control and post‐translational mechanisms [6]. A number of transcription factors have been identified as regulators of PD‐L1 transcription, influencing cancer immunity during cancer progression or therapy [6]. For example, ATF3 acts as a transcriptional activator of PD‐L1 expression; its inhibition led to decreased PD‐L1 levels and enhanced the population of activated tumour‐infiltrating CD8+ T cells in melanoma models [26]. FoxO3a, a member of the FoxO transcription factor family, is widely expressed at varying levels and regulates the expression of target genes involved in cancer cell cycle progression, apoptosis, metastasis, angiogenesis, and metabolism through direct binding to gene promoters or interaction with other regulatory factors [24, 27]. MAPK and PI3K serve as upstream regulators of FoxO3a and have been implicated in the regulation of PD‐L1 expression, as reported previously [28]. This association suggests a potential connection between FoxO3a and PD‐L1. In this study, we found that FoxO3a was a direct positive regulator of PD‐L1 transcription. Specifically, we observed that FoxO3a enhanced the protein levels of PD‐L1 in TNBC cells, thereby promoting cancer cell immune evasion. The findings into the modulation of PD‐L1 mRNA levels by FoxO3a, along with luciferase assays examining the promoter activity of the PD‐L1 gene, confirmed that FoxO3a exerted translational control over PD‐L1 expression levels in TNBC. Furthermore, our site‐mutation analysis of the gene promoter and subsequent ChIP‐PCR results support the direct binding of FoxO3a to the PD‐L1 gene promoter region, thus enhancing promoter activity in TNBC. Therefore, our results demonstrated that FoxO3a plays a direct role in regulating PD‐L1 transcriptional expression in TNBC, providing new insights into FoxO3a's function as a tumour promoter by facilitating TNBC cell immune evasion.

Although monoclonal antibodies are the most common PD1/PD‐L1 blocking agents, research and development of small‐molecule inhibitors targeting the PD1/PD‐L1 pathway are underway [29]. An example is metformin, a drug used clinically to treat type 2 diabetes, which has shown promising results in regulating PD‐L1 expression [30]. Metformin decreased the expression of PD‐L1 in cancer cells by promoting abnormal PD‐L1 glycosylation and degradation, therefore blocking immune‐inhibitory signalling and enhancing antitumor immunity. In this study, we revealed that DA, a clinically established antimalarial drug known for its safety and affordability, holds potential as a therapeutic agent for inhibiting tumour growth and enhancing antitumor immunity in TNBC numerous studies have shown that targeting the proliferation, migration, or metastasis of breast cancer is an effective strategy for screening potential therapeutic drugs [31, 32]. The mechanism of artemisinin and DA in antimalaria treatment involves inducing oxidative stress [33]. In our study, we found that DA promoted cell death and inhibited tumour growth in TNBC, supporting previous research on the anticancer effects of DA, which has primarily focused on inducing cancer cell apoptosis, inhibiting metastasis and overcoming chemotherapy resistance [34, 35]. Recent studies have also shown DA's ability to modulate immune cell populations within the tumour microenvironment. In vitro studies have shown that DA promoted the proliferation of Treg cells while inhibiting the differentiation of Th1 and Th17 cells [36]. In an orthotopic pancreatic cancer model, DA suppressed the expansion of M2‐like tumour‐associated macrophages and myeloid‐derived suppressor cells, while enhancing the populations of CD8+ T cells, NK cells and NKT cells in tumour tissues [37]. Our findings present new evidence supporting DA as a potential therapeutic agent for TNBC, targeting PD‐L1 expression. We demonstrated that DA inhibited PD‐L1 mRNA and protein levels in human TNBC cells in vitro and in a nude mouse tumour model. Functionally, the suppression of PD‐L1 expression in DA‐treated MDA‐MB‐231 cells led to reduced PD1 protein binding to cancer cells and enhanced killing of activated T cells against cancer cells, indicating an inhibition of PD‐L1 and tumour immune evasion by DA. Consistent with our expectations, FoxO3a is necessary for DA inhibiting PD‐L1 expression in TNBC. We found a decrease in FoxO3a levels in DA‐treated TNBC cells. Moreover, DA treatment reduced PD‐L1 gene promoter activity in MDA‐MB‐231 cells, which necessitated the presence of the FoxO3a binding site s155 element within the PD‐L1 gene promoter. These findings revealed that DA has the potential to suppress PD‐L1 expression, thereby promoting antitumor immunity, through inhibition of FoxO3a and its binding to the PD‐L1 gene promoter.

Phosphorylation of FoxO3a is important in regulating its cytoplasmic‐nuclear localization, transcriptional activity and protein stability [38]. PI3K/AKT‐mediated phosphorylation of FoxO3a leads to its association with 14‐3‐3 proteins, retaining it in the cytoplasm [13]. IKK‐mediated phosphorylation of FoxO3a at Ser644 promotes its nuclear export and subsequent proteasomal degradation via ubiquitination [14]. In this study, we found that DA treatment not only increased IKK phosphorylation but also enhanced FoxO3a phosphorylation at Ser644 and its ubiquitination, indicating a role for DA in driving FoxO3a degradation in MDA‐MB‐231 cells. The ER stress response involves three signalling branches: IRE1α, ATF6 and PERK [39]. In response to ER stress, phosphorylated IRE1 interacts with TRAF2, which in turn leads to the phosphorylation and activation of IKK [25]. DA has been reported to activate ER stress‐related proteins such as GRP78, endoplasmic reticulum calcium ATPase, PERK, IRE1 and ATF6 in both liver and colorectal cancer cells [35]. Consistent with these findings, our results show that in TNBC cells, DA treatment activates IRE1 while having no apparent effect on ATF6 and PERK. Moreover, DA treatment enhances the protein interactions among IRE1, IKK and TRAF2. These observations suggest a mechanism wherein DA‐induced ER stress, mediated predominantly through IRE1, promotes IKK activation. This cascade subsequently drives FoxO3a phosphorylation at Ser644, resulting in its ubiquitin‐mediated degradation.

In summary, our study identified PD‐L1 as a direct transcriptional target of FoxO3a in TNBC, demonstrating that FoxO3a positively regulates PD‐L1 expression to promote tumour immune evasion. Moreover, we found that DA treatment effectively downregulated PD‐L1 expression, thereby enhancing T‐cell‐mediated antitumor immunity against TNBC cells. This effect was, at least in part, mediated through the activation of the IRE1/IKK/FoxO3a signalling cascade. Given the favourable safety profile and cost‐effectiveness of DA, our findings suggest its potential therapeutic utility in managing TNBC. Future studies will further elucidate the mechanisms by which DA and its derivatives exert antitumor effects, potentially guiding the development of novel therapeutic strategies for TNBC and other cancers.

## Author Contributions

**Xingan Xing:** conceptualization (equal), formal analysis (equal), investigation (equal), visualization (equal), writing – original draft (equal). **Zhiwei Zhou:** methodology (equal), resources (equal). **Mohd Farhan:** methodology (equal), visualization (equal). **Xia Zhao:** formal analysis (equal), software (equal). **Shuai Li:** methodology (equal), software (equal). **Bingxi Lei:** investigation (equal), writing – review and editing (equal). **Jiankang Fang:** resources (equal), writing – review and editing (equal). **Wenshu Zhou:** software (equal). **Wenhua Zheng:** conceptualization (equal), funding acquisition (equal), project administration (equal), writing – review and editing (equal).

## Ethics Statement

The animal study was reviewed and approved by the Ethics Committee of the University of Macau (protocol No.: UMARE‐015‐2017).

## Conflicts of Interest

The authors declare no conflicts of interest.

## Data Availability

The data that support the findings of this study are available from the corresponding author upon reasonable request.
